# Psychosocial and Behavioral Impact of COVID-19 in Autism Spectrum Disorder: An Online Parent Survey

**DOI:** 10.3390/brainsci10060341

**Published:** 2020-06-03

**Authors:** Marco Colizzi, Elena Sironi, Federico Antonini, Marco Luigi Ciceri, Chiara Bovo, Leonardo Zoccante

**Affiliations:** 1 Section of Psychiatry, Department of Neurosciences, Biomedicine and Movement Sciences, University of Verona, 37134 Verona, Italy; 2 Department of Psychosis Studies, Institute of Psychiatry, Psychology and Neuroscience, King’s College London, London SE5 8AF, UK; 3 Child and Adolescent Neuropsychiatry Unit, Maternal-Child Integrated Care Department, Integrated University Hospital of Verona, 37126 Verona, Italy; sironi.elena@yahoo.it (E.S.); fede187ant@gmail.com (F.A.); marcoluigi.ciceri@aovr.veneto.it (M.L.C.); leonardo.zoccante@aovr.veneto.it (L.Z.); 4 Medical Direction, Integrated University Hospital of Verona, 37126 Verona, Italy; direzione.sanitaria@aovr.veneto.it

**Keywords:** coronavirus, 2019-nCoV, neurodevelopment, child and adolescent psychiatry, mental health prevention

## Abstract

The 2019 coronavirus disease (COVID-19) outbreak could result in higher levels of psychological distress, especially among people suffering from pre-existing mental health conditions. Young individuals with autism spectrum disorders (ASD) are particularly at risk due to their vulnerability to unpredictable and complex changes. This study aimed to investigate the impact of the COVID-19 pandemic on ASD individuals, whether any pre-pandemic sociodemographic or clinical characteristics would predict a negative outcome, and to narratively characterize their needs. Parents and guardians of ASD individuals filled out an online survey consisting of 40 questions investigating socio-demographic and clinical characteristics of their children, impact of the COVID-19 outbreak on their wellbeing and needs to deal with the emergency. Data were available on 527 survey participants. The COVID-19 emergency resulted in a challenging period for 93.9% of families, increased difficulties in managing daily activities, especially free time (78.1%) and structured activities (75.7%), and, respectively, 35.5% and 41.5% of children presenting with more intense and more frequent behavior problems. Behavior problems predating the COVID-19 outbreak predicted a higher risk of more intense (odds ratio (OR) = 2.16, 95% confidence interval (CI) 1.42–3.29) and more frequent (OR = 1.67, 95% CI 1.13–2.48) disruptive behavior. Even though ASD children were receiving different types of support, also requiring specialist (19.1%) or emergency (1.5%) interventions in a relatively low proportion of cases, a number of needs emerged, including receiving more healthcare support (47.4%), especially in-home support (29.9%), as well as interventions to tackle a potentially disruptive quarantine (16.8%). The COVID-19 outbreak has undoubtedly resulted in increased difficulties among ASD individuals.

## 1. Introduction

After the Severe Acute Respiratory Syndrome (SARS) Coronavirus outbreak of 2002–2003, the International Health Regulations (IHR) of the World Health Organization (WHO), which had been first adopted in 1969, were revised in 2005 to extend their scope to any public health risk that might affect human health, irrespective of the source. Emphasis was put on the risk that the increasing international travel and trade could facilitate the international spread of disease, requiring a coordinated international response. Since the 2005 IHR adoption, the WHO has formally declared six Public Health Emergencies of International Concern (PHEIC), the latter of which, the 2019 coronavirus disease (COVID-19), is still ongoing [1]. COVID-19 is caused by a newly identified coronavirus which can induce SARS in man (SARS-CoV-2), as a consequence of a probable zoonotic spillover [2], firstly reported in Central China in December 2019 [3]. Due to person-to-person transmission, it has rapidly spread in Europe [4], with northern Italy becoming Europe’s epicenter [5], and USA [6]. As of 1 May 2020, over 3 million cases have been reported worldwide, affecting more than 200 countries.

Since the beginning of the pandemic, most clinical and research efforts have been allocated to advance our understanding of the virus properties and pathogenic armory in order to treat the infection and protect from it [7]. However, according to some research evidence, the COVID-19 pandemic is also unraveling a potential gap in mental health services during emergencies [8]. In particular, the COVID-19 outbreak would result in higher levels of psychological distress among the general population [8] as well as a higher risk or symptom exacerbation among people suffering from a pre-existing mental health condition [9], possibly triggered by concerns about its rapid escalation and global spread [1] as a deadly threat [10]. Furthermore, alarming media reports may unintendedly amplify fear reactions [11], with potential detrimental consequences for people susceptible to negative emotional states. Importantly, the pandemic has required unprecedented measures by national governments including imposing quarantine to citizens [12]. The experience of being quarantined may be negative, as evidence suggests a wide range of long-lasting mental health problems in a substantial proportion of individuals [13]. While there is no strong evidence that any particular demographic factors carry a higher risk of poor psychological outcome following the obligation of home quarantine [13], a pre-existing psychiatric history seems to predict a worse outcome [14] and a higher need for support during quarantine [13]. 

Among vulnerable populations, young individuals with autism spectrum disorders (ASD) are of particular concern for the impact that the COVID-19 outbreak may have on their wellbeing as well as the specific support they may need to preserve their mental health through the pandemic [15]. ASD are a group of conditions characterized by social communication problems, difficulties with reciprocal social interactions, and unusual patterns of repetitive behavior [16]. Such features are associated with a preference for highly predictable environments, whereas ASD individuals may feel stressed, anxious or confused if unpredictable or complex changes occur [17]. The COVID-19 outbreak has undoubtedly led to a quick-paced and rapidly shifting social situation which may increase ASD individuals’ difficulties.

The purpose of this study was threefold. The main aim was to rapidly investigate the impact of the COVID-19 outbreak on ASD individuals through an online parent survey carried in Northern Italy, one of the European regions mostly affected [5]. Due to the ASD individuals’ difficulty to deal with the unexpected, a mainly unfavorable psychosocial and behavioral outcome was hypothesized. A further aim was to investigate whether any pre-pandemic sociodemographic or clinical characteristics would predict a negative impact of the pandemic on ASD individuals’ wellbeing. Based on previous evidence [13,14], psychological problems predating the emergency were hypothesized to predict a poor outcome. Finally, the survey served to characterize the needs of ASD individuals and their families from a narrative perspective, by collecting the parents’ perceptions, as a first step to improving their quality of health care.

## 2. Materials and Methods

### 2.1. Research Design

Google Forms was used to create an online parent survey to be shared through the dissemination of a hyperlink. The survey was available online from the 6 April to the 20 April 2020. All participants provided electronic informed consent that contained information about the purpose of the study, procedures, benefits of participating, voluntary participation, and contact information of the researchers. The survey was part of a larger study which was approved by the research ethics committee at the Integrated University Hospital of Verona (CESC 2242 and CESC 2243). 

### 2.2. Participants

Parents and guardians of individuals with an ASD diagnosis were asked by healthcare professionals affiliated with the Veneto Autism Spectrum Disorder Regional Centre at the Integrated University Hospital of Verona to fill out the online survey. Autism advocacy and family support networks were additionally used to distribute and directly encourage survey participation. Children’ ASD diagnosis was self-reported.

### 2.3. Instrument

The parent survey was developed by a focus group of physicians, psychologists, and child life specialists, also taking the advice from parents of children with ASD. The survey consisted of 40 questions (18 multiple choice questions, 20 yes/no questions, and 2 open-response questions) in 3 categories: (i) ASD individuals’ socio-demographic and clinical characteristics, (ii) impact of the COVID-19 outbreak on their wellbeing, and (iii) needs to deal with the emergency. Participants were allowed to select only 1 item for each multiple-choice question. 

### 2.4. Analyses

The final raw data were downloaded from Google Forms into a Microsoft Excel file for analysis using SPSS software (Version 26.0; IBM Corp, Armonk, NY, USA). Descriptive statistics were used to provide baseline information concerning survey participants’ ASD children. Then, multiple logistic regressions were performed to investigate whether any ASD individuals’ socio-demographic or clinical characteristics would predict a greater frequency and intensity of behavior problems following the COVID-19 outbreak. 

The open-response questions did not have a scoring system. The needs identified by the survey responses were gathered with the intent of informing healthcare professionals in their assessment and management of individuals with an ASD diagnosis during the ongoing COVID-19 emergency. Two authors independently evaluated such answers and pooled them into categories (e.g., healthcare, social, financial needs, etc.). In the rare instances of discrepant category attribution, consensus was reached through discussion with a third senior clinical researcher.

The survey was not intended to formally assess severity of ASD among participants’ children.

## 3. Results

A total of 529 respondents participated in the survey. As 2 participants did not answer any questions at all, a total of 527 participants were included in the study. Across the 38 closed questions, survey participants provided 18,738 answers, while 373 answers were missing (<2%). Further, 34 answers and 1 missing item were deemed inconsistent (<0.2%) and excluded from the final analyses.

### 3.1. Socio-Demographic and Clinical Characteristics

The mean age of participants’ children was 13 years (SD = 8.1). Almost all of them were from the Veneto region (99.4%) and the large majority were living in married or cohabiting couple families (88.2%). Most children had at least 1 sibling (71.6%), and one out every 10 siblings had a neurodevelopmental disorder (NDD) diagnosis (10.2%). Most children were receiving private therapy (66.2%) and most parents were members of autism advocacy and family support networks (67.1%) (Table 1). 

Only one every three children had a fluent language (33.1%). About half of the entire sample was presenting with behavior problems from before the outbreak of COVID-19 (51.5%); among those, 42.2% were receiving pharmacological treatment. At least 1 comorbid medical condition was reported in 27.8% of ASD individuals, with neuromotor and gastrointestinal conditions and allergies and food sensitivity being the conditions most frequently reported (Table 1 and Supplementary Table S1). 

### 3.2. Psychosocial and Behavioral Impact of the Emergency Outbreak

COVID-19 positivity was reported among 1.3% of nuclear family members and 4.4% of extended family members, with bereavement occurring in 2.3% of cases. Approximately one out of four parents stopped working due the emergency outbreak (26.1% of mothers and 27.5% of fathers). The large majority of them evaluated the current period of change and restrictions as challenging or very challenging (93.9%) and more challenging than before the emergency outbreak (77%) (Table 2).

Following the emergency outbreak, a proportion of parents reported support from the Local Healthcare Services (27.7%), with the large majority of them reporting both direct (70.1%; e.g., calls, videocalls) and indirect (84%; e.g., text messages, homework assignments) school support as well as support from the private therapist (73.3%). For each type of support, an only slightly lower proportion of parents considered it from sufficiently useful to very useful during the ongoing emergency (Table 2).

A proportion of parents reported difficulties in managing their child’s meals (23%), autonomies (31%), free time (78.1%), and structured activities (75.7%). For each activity, an almost overlapping proportion of parents reported such activity as more difficult than before the emergency outbreak. Overall, compared to before the emergency outbreak, behavior problems were reported being more intense (35.5%) and more frequent (41.5%) in a substantial proportion of ASD individuals. Due to the onset of behavior problems, an emergency contact with the child’s Neuropsychiatrist was required in 19.1% of cases, while an access to the Accident and Emergency (A&E) happened in 1.5% of cases (Table 2).

### 3.3. Predictors of Emergency Outbreak Negative Impact on Wellbeing

A multiple logistic regression tested for an effect of (i) behavior problems predating the emergency (yes/no), (ii) age, (iii) language (fluent/non-fluent), (iv) being an only child (yes/no) as a proxy of greater social isolation in quarantine, (v) medical comorbidity (yes/no), (vi) parenting couple situation (married or cohabiting/separated or single parent), (vii) support by Local Healthcare Services since COVID-19 (yes/no), (viii) direct school support since COVID-19 (yes/no), (ix) indirect school support since COVID-19 (yes/no), (x) private therapist support since COVID-19 (yes/no or no private therapist from before COVID-19) on the intensity of the behavior problems following the emergency outbreak (more intense/equally or less intense). The logistic regression model was statistically significant, *χ*^2^ (10, N = 440) = 32.338, *p* < 0.001. ASD individuals with preexisting behavior problems were 2.16 times more likely to exhibit more intense behavior problems that those without preexisting behavior problems. Increasing age and living with a separated or single parent were associated with a reduction in the likelihood of exhibiting more intense behavior problems, while not receiving indirect school support during the emergency tended to be associated with an increased likelihood of exhibiting more intense behavior problems (Table 3).

A further multiple logistic regression tested for the effect of (i) behavior problems predating the emergency (yes/no), (ii) age, (iii) language (fluent/non-fluent), (iv) being an only child (yes/no), (v) medical comorbidity (yes/no), (vi) parenting couple situation (married or cohabiting/separated or single parent), (vii) support by Local Healthcare Services since COVID-19 (yes/no), (viii) direct school support since COVID-19 (yes/no), (ix) indirect school support since COVID-19 (yes/no), (x) private therapist support since COVID-19 (yes/no or no private therapist from before COVID-19) on the frequency of the behavior problems following the emergency outbreak (more frequent/equally or less frequent). The logistic regression model was statistically significant, *χ*^2^ (10, N = 444) = 18.502, *p* = 0.047. ASD individuals with preexisting behavior problems were 1.67 times more likely to exhibit more frequent behavior problems that those without preexisting behavior problems (Table 4).

### 3.4. Needs to Deal with the Emergency: A Narrative Perspective

Out of 527 survey participants, 406 parents (77%) reported at least one need to the open-response question about what could be of help do deal with the ongoing emergency. Ten of them reported more than one need (4 participants reported 2 needs, 6 participants reported 3 needs), for a total of 422 responses. The most commonly reported need was for in-home healthcare support (29.9%), followed by center-based healthcare support (10.4%), loosening quarantine restrictions (9.7%), ending lockdown (7.1%), and in-hospital healthcare support (7.1%; Table 5).

## 4. Discussion

To our knowledge, this is the first study which systematically explored the impact of the COVID-19 outbreak in a population of individuals suffering from an autism spectrum disorder (ASD). Results from this parent survey indicate that the large majority of parents of ASD individuals consider the period of change and restrictions that has followed the onset of the emergency as challenging and requiring more commitment than before. Most support was delivered by school services, followed by private therapists and local healthcare services. Consistent with previous reports of executive functioning deficits making ASD individuals more vulnerable to routine disruption [18], an elevated number of parents reported difficulties in managing their children’s daily activities, especially in terms of free time and structured activities. Despite requiring specialist intervention in a relatively small proportion of cases, and almost never ending in hospital emergency assessments, behavior problems worsened in more than one third of ASD individuals. 

In line with previous evidence that pre-existing psychological difficulties seem to be the only clear predictor of poorer mental health outcomes following respiratory syndromes [14] and quarantine [13], the most relevant finding of this survey is that ASD individuals with behavior problems predating the COVID-19 outbreak are twice as likely to experience more intense and more frequent behavior problems since the beginning of the emergency. As independent evidence supports decreasing symptom levels from childhood to young adulthood in ASD, especially in verbally fluent individuals [19], we also tested whether the age and language level of survey participants’ children with ASD would predict a worsening of the behavior problems. Results suggest that older age may play a protective role with regards to the emergency-induced intensification of behavior problems, while the effect of language failed to reach statistical significance. Furthermore, despite social isolation and mental health problems may co-occur in childhood [20], as well as medical conditions and ASD [21], being an only child, as a proxy of greater social isolation, and comorbid medical conditions did not predict a poorer outcome in terms of more intense or more frequent behavior problems following the COVID-19 outbreak and the implementation of restrictive measures and quarantine. Interestingly, living with a separated or single parent was associated with a better outcome in terms of intensity of behavior problems. Despite being counterintuitive, such a finding may reflect a more simplified parent–child interaction which could be effective in preventing the deterioration of the child’s wellbeing during quarantine and restrictions. Future studies would need to clarify this issue. Furthermore, it is important to highlight that such evidence does not necessarily apply to other social contexts. Finally, ASD individuals not receiving school support since the COVID-19 outbreak tended to express more intense behavior problems, suggesting the importance of maintaining contact with the school during the emergency.

The last scope of this study was to narratively collect the parents’ perceived needs through the emergency by offering an open-response question at the end of the survey. About half of the participants reported needing support from healthcare services, especially in-home services. Interestingly, almost one every five parents reported that loosening restrictions or ending the lockdown would be of help.

Outbreaks of emerging infections such as COVID-19 can elicit strong fear reactions and preoccupations with downstream effects on physical and mental health, especially in vulnerable individuals [22]. Moreover, such negative impacts on health could be worsened by the experience of being quarantined [13]. Autism is no exception. Even though it is a complex genetic disorder, the effect of the environment in shaping the behavioral phenotype should not be underestimated. In fact, a high emotional climate, such as that resulting from the COVID-19 emergency outbreak, has been associated with increased levels of maladaptive behavior in ASD over time [23]. Furthermore, families of individuals with ASD have been reported to experience greater stress than families whose children suffer from other disabilities [24,25], making a compelling case for the implementation of youth-oriented mental health prevention and early intervention strategies [26,27]. Evidence supports the effectiveness of such interventions in mitigating disabilities and even improving skills among young individuals with ASD [28].

Surveys performed during previous Public Health Emergencies of International Concern (PHEIC) have provided timely data to inform best practices in responding to the emergency [29]. Similarly, the online survey presented here has proved to be a powerful data collection tool, benefiting from the strengths that have been associated to this type of instrument such as having a large sample size, fast response times, timely data processing, and low costs [30]. Moreover, thanks to the solid infrastructure of autism advocacy and family support networks, we were able to mitigate the risk of poor response rates and improve sample representativeness, collecting data from over five hundred ASD individuals and their families in a catchment area (the Veneto Region) of about 5 million people. However, the findings of this study have to be seen in light of some limitations. In particular, in spite of the aforementioned advantages, the survey suffered from the lack of a standardized assessment of clinical features such as language and behavior problems. Such aspects may limit the comparability of outcomes among different studies as well as internal comparisons in any follow-up assessments. Furthermore, despite collecting information on children’s language and behavior problems predating the emergency as a proxy of their baseline cognitive and adaptive functioning, due to its nature, the online survey did not allow investigating such aspects through a standard method such as psychometric tests. Furthermore, information on the gender of ASD children was not available through the online survey. Even though the main aim of the study was not to investigate gender differences in response to the COVID-19 outbreak in ASD, we acknowledge the limitation. 

In conclusion, the present survey indicates that the ongoing COVID-19 emergency has resulted in a challenging period for most ASD individuals and their families, with increased difficulties in managing daily activities and at least one in every three children presenting with more frequent or more intense behavior problems. Children with behavior problems predating the COVID-19 outbreak were found to be particularly at risk to present with more intense and more frequent disruptive behavior. Even though ASD children were receiving different types of support, also requiring specialist or emergency interventions in a relatively low proportion of cases, a number of needs emerged, including receiving more healthcare support, especially from in-home services, as well as interventions to tackle a potentially disruptive quarantine.

## Figures and Tables

**Table 1:** Socio-demographic and clinical characteristics.

	M	(SD)
Age (years)	13	8.1
	**N**	**(%)**
Veneto Region Province		
Belluno	41	7.8
Padova	88	16.8
Rovigo	26	5
Treviso	40	7.6
Venezia	73	13.9
Verona	149	28.4
Vicenza	105	20
Other Region	3	0.6
Missing	2	
Parenting couple situation		
Married/Cohabiting	463	88.2
Separated	31	5.9
Single parent	31	5.9
Missing	2	
Only child		
No	374	71.6
Yes	148	28.4
Missing	2	
Number of siblings *		
1	290	77.5
2	65	17.4
3	15	4
4	3	0.8
5	1	0,3
Missing	0	
Siblings diagnosed with NDD (ASD, ADHD, etc.) *		
No	336	89.8
Yes	38	10.2
Missing	0	
Child receiving private therapy		
No	175	33.8
Yes	342	66.2
Missing	4	
Membership in Autism advocacy/family support networks		
No	172	32.9
Yes	351	67.1
Missing	4	
Child’s language level		
Fluent speech	174	33.1
Phrase speech	146	27.8
No phrase speech	205	39
Missing	2	
The child was presenting with behavior problems from before COVID-19		
No	251	48.5
Yes	266	51.5
Missing	6	
Pharmacological treatment for behavior problems **		
No	152	57.8
Yes	111	42.2
Missing	3	
Comorbid medical conditions		
No	377	72.2
Yes	145	27.8
Missing	5	

* Of those reporting siblings; ** Of those reporting behavior problems from before COVID-19; NDD, Neurodevelopment disorder; ASD, Autism spectrum disorder; ADHD, Attention Deficit Hyperactivity Disorder.

**Table 2:** Psychosocial and behavioral impact of the emergency outbreak.

	N	(%)
COVID-19 positivity among nuclear family members		
No	519	98.7
Yes	7	1.3
Missing	1	
COVID-19 positivity among extended family members		
No	500	95.6
Yes	23	4.4
Missing	4	
Bereavement due to COVID-19		
No	514	97.7
Yes	12	2.3
Missing	1	
Mother’s current working situation		
Regularly commuting to work	92	17.6
Smart working	98	18.7
Not working because of COVID-19	137	26.1
Not working since before COVID-19	197	37.6
Missing	3	
Father’s current working situation		
Regularly commuting to work	212	42
Smart working	104	20.6
Not working because of COVID-19	139	27.5
Not working since before COVID-19	50	9.9
Missing	22	
Judgement on this period of change and restrictions		
Very challenging	284	54
Challenging	210	39.9
Not challenging	32	6.1
*Missing*	1	
Judgement on this period of change and restrictions as compared to before COVID-19		
More challenging	405	77
Equally challenging	86	16.3
Less challenging	35	6.7
Missing	1	
Support by Local Healthcare Services since COVID-19		
Daily contacts	7	1.4
Weekly contacts	98	19.4
Twice weekly contacts	35	6.9
No contact	366	72.3
Missing	21	
Usefulness of support by Local Healthcare Services during COVID-19		
Very useful	10	2.2
Useful	28	6.1
Sufficiently useful	65	14.1
Not very useful	93	20.1
Not useful	266	57.6
Missing	65	
Direct school support since COVID-19		
Daily contacts	110	22.5
Weekly contacts	157	32.2
Twice weekly contacts	75	15.4
No contact	146	29.9
Missing	39	
Indirect school support since COVID-19		
Daily contacts	138	28.7
Weekly contacts	159	33.1
Twice weekly contacts	106	22.1
No contact	77	16
Missing	47	
Usefulness of school support during COVID-19		
Very useful	60	12.9
Useful	113	24.4
Sufficiently useful	116	25
Not very useful	93	20
Not useful	82	17.7
Missing	63	
Private therapist support since COVID-19		
Daily contacts	43	12.6
Weekly contacts	148	43.4
Twice weekly contacts	59	17.3
No contact	91	26.7
Missing	1	
Usefulness of private therapist during COVID-19		
Very useful	65	19.6
Useful	80	24.1
Sufficiently useful	64	19.3
Not very useful	50	15.1
Not useful	73	22
Missing	10	
Difficulties in managing the child’s meals since COVID-19		
No	404	77
Yes	121	23
Missing	2	
Greater difficulties in managing the child’s meals as compared to before COVID-19		
No	378	71.9
Yes	148	28.1
Missing	1	
Difficulties in managing the child’s autonomies since COVID-19		
No	361	69
Yes	162	31
Missing	4	
Greater difficulties in managing the child’s autonomies as compared to before COVID-19		
No	372	71
Yes	152	29
Missing	3	
Difficulties in managing the child’s free time since COVID-19		
No	115	21.9
Yes	411	78.1
Missing	1	
Greater difficulties in managing the child’s free time as compared to before COVID-19		
No	97	18.4
Yes	429	81.6
Missing	1	
Difficulties in managing the child’s structured activities since COVID-19		
No	126	24.3
Yes	393	75.7
Missing	8	
Greater difficulties in managing the child’s structured activities as compared to before COVID-19		
No	123	23.8
Yes	394	76.2
Missing	10	
Intensity of the child’s behavior problems as compared to before COVID-19		
More intense	183	35.5
Equally intense	264	51.3
Less intense	68	13.2
Missing	12	
Frequency of the child’s behavior problems as compared to before COVID-19		
More frequent	216	41.5
Equally frequent	229	44
Less frequent	76	14.6
Missing	6	
Contacts with the child’s Neuropsychiatrist due to behavioral problems since COVID-19		
No	424	80.9
Yes	100	19.1
Missing	3	
Access to A&E for child’s behavioral problems since COVID-19		
No	514	98.5
Yes	8	1.5
Missing	5	

**Table 3:** Predictors of emergency outbreak negative impact on intensity of behavior problems.

	B	S.E.	Wald Chi-Square	*p*-Value	OR	95% CI
Age	−0.037	0.019	3.981	0.046	0.963	0.929–0.999
Behavior problems predating COVID-19	0.770	0.215	12.869	<0.001	2.160	1.418–3.291
Non-fluent language	0.352	0.243	2.100	0.147	1.422	0.883–2.290
Medical comorbidity	0.317	0.241	1.726	0.189	1.372	0.856–2.201
Only child	0.286	0.233	1.507	0.220	1.331	0.843–2.102
Separated/single parent	−0.778	0.383	4.127	0.042	0.459	0.217–0.973
No support from Local Health Service	0.050	0.241	0.043	0.836	1.051	0.656–1.685
No direct support from school	−0.127	0.255	0.247	0.619	0.881	0.534–1.453
No indirect support from school	0.605	0.322	3.540	0.060	1.831	0.975–3.439
No support from private therapist	−0.073	0.214	0.118	0.732	0.929	0.610–1.414

Note: OR, odds ratio; CI, confidence interval.

**Table 4:** Predictors of emergency outbreak negative impact on frequency of behavior problems.

	B	S.E.	Wald Chi-Square	*p*-Value	OR	95% CI
Age	−0.024	0.018	1.841	0.175	0.976	0.943–1.011
Behavior problems predating COVID-19	0.513	0.201	6.509	0.011	1.670	1.126–2.477
Non-fluent language	0.304	0.226	1.800	0.180	1.355	0.869–2.111
Medical comorbidity	0.221	0.229	0.933	0.334	1.248	0.796–1.954
Only child	−0.004	0.224	0.000	0.984	0.996	0.642–1.544
Separated/ single parent	−0.229	0.329	0.486	0.486	0.795	0.417–1.515
No support from Local Health Service	−0.312	0.227	1.888	0.169	0.732	0.469–1.142
No direct support from school	−0.334	0.246	1.849	0.174	0.716	0.442–1.159
No indirect support from school	0.406	0.312	1.694	0.193	1.501	0.814–2.769
No support from private therapist	−0.137	0.203	0.455	0.500	0.872	0.586–1.298

Note: OR, odds ratio; CI, confidence interval.

**Table 5:** Responses to the open-response question about what could be of help do deal with the ongoing emergency.

	N	%
In-home healthcare support	126	29.9
Center-based healthcare support	44	10.4
Loosening quarantine restrictions	41	9.7
Ending lockdown	30	7.1
In-hospital healthcare support	30	7.1
Increase school support	29	6.9
Help setting a daily schedule	21	5
“Nothing”	20	4.7
“Don’t know”	14	3.3
Parent support	13	3.1
Peer relationship	12	2.8
Structured physical activity	9	2.1
Community support	9	2.1
Financial family support	8	1.9
Work support	6	1.4
Spiritual and religious reflections	4	0.9
Information technology support	3	0.7
Pharmacological support	3	0.7

## Supplementary Materials

The following are available online at https://www.mdpi.com/2076-3425/10/6/341/s1. Table S1: Responses to the open-response question about what medical comorbidity was present in children with ASD.

## References

[1] Cucinotta D., Vanelli M. (2020). WHO Declares COVID-19 a Pandemic. Acta Biomed..

[2] Lu R., Zhao X., Li J., Niu P., Yang B., Wu H., Wang W., Song H., Huang B., Zhu N. (2020). Genomic characterisation and epidemiology of 2019 novel coronavirus: Implications for virus origins and receptor binding. Lancet.

[3] Li Q., Guan X., Wu P., Wang X., Zhou L., Tong Y., Ren R., Leung K.S.M., Lau E.H.Y., Wong J.Y. (2020). Early Transmission Dynamics in Wuhan, China, of Novel Coronavirus-Infected Pneumonia. N. Engl. J. Med..

[4] Rothe C., Schunk M., Sothmann P., Bretzel G., Froeschl G., Wallrauch C., Zimmer T., Thiel V., Janke C., Guggemos W. (2020). Transmission of 2019-nCoV Infection from an Asymptomatic Contact in Germany. N. Engl. J. Med..

[5] Gagliano A., Villani P.G., Cò F.M., Paglia S., Bisagni P.A.G., Perotti G.M., Storti E., Lombardo M. (2020). 2019-ncov’s epidemic in middle province of northern Italy: Impact, logistic & strategy in the first line hospital. Disaster Med. Public Health Prep..

[6] Ghinai I., McPherson T., Hunter J., Kirking H., Christiansen D., Joshi K., Rubin R., Morales-Estrada S., Black S., Pacilli M. (2020). First known person-to-person transmission of severe acute respiratory syndrome coronavirus 2 (SARS-CoV-2) in the USA. Lancet.

[7] Berger Z.D., Evans N.G., Phelan A.L., Silverman R.D. (2020). Covid-19: Control measures must be equitable and inclusive. BMJ.

[8] Lima C.K.T., Carvalho P.M.M., Lima I.A.A.S., Nunes J.V.A.O., Saraiva J.S., de Souza R.I., da Silva C.G.L., Neto M.L.R. (2020). The emotional impact of Coronavirus 2019-nCoV (new Coronavirus disease). Psychiatry Res..

[9] Yao H., Chen J.H., Xu Y.F. (2020). Patients with mental health disorders in the COVID-19 epidemic. Lancet Psychiatry.

[10] Onder G., Rezza G., Brusaferro S. (2020). Case-Fatality Rate and Characteristics of Patients Dying in Relation to COVID-19 in Italy. JAMA.

[11] Garfin D.R., Silver R.C., Holman E.A. (2020). The novel coronavirus (COVID-2019) outbreak: Amplification of public health consequences by media exposure. Health Psychol..

[12] Wilder-Smith A., Freedman D.O. (2020). Isolation, quarantine, social distancing and community containment: Pivotal role for old-style public health measures in the novel coronavirus (2019-nCoV) outbreak. J. Travel Med..

[13] Brooks S.K., Webster R.K., Smith L.E., Woodland L., Wessely S., Greenberg N., Rubin G.J. (2020). The psychological impact of quarantine and how to reduce it: Rapid review of the evidence. Lancet.

[14] Jeong H., Yim H.W., Song Y.J., Ki M., Min J.A., Cho J., Chae J.H. (2016). Mental health status of people isolated due to Middle East Respiratory Syndrome. Epidemiol. Health.

[15] Narzisi A. (2020). Handle the Autism Spectrum Condition During Coronavirus (COVID-19). Brain Sci..

[16] American Psychiatric Publishing (2013). Diagnostic and Statistical Manual of Mental Disorders.

[17] Baron-Cohen S. (2006). The hyper-systemizing, assortative mating theory of autism. Prog. Neuropsychopharmacol. Biol. Psychiatry.

[18] Narzisi A., Muratori F., Calderoni S., Fabbro F., Urgesi C. (2013). Neuropsychological Profile in High Functioning Autism Spectrum Disorders. J. Autism Dev. Disord..

[19] Bal V.H., Kim S.H., Fok M., Lord C. (2019). Autism spectrum disorder symptoms from ages 2 to 19 years: Implications for diagnosing adolescents and young adults. Autism Res..

[20] Matthews T., Danese A., Wertz J., Ambler A., Kelly M., Diver A., Caspi A., Moffitt T.E., Arseneault L. (2015). Social isolation and mental health at primary and secondary school entry: A longitudinal cohort study. J. Am. Acad. Child Adolesc. Psychiatry.

[21] Tye C., Runicles A., Whitehouse A., Alvares G. (2019). Characterizing the Interplay Between Autism Spectrum Disorder and Comorbid Medical Conditions: An Integrative Review. Front. Psychiatry.

[22] Colizzi M., Bortoletto R., Silvestri M., Mondini F., Puttini E., Cainelli C., Gaudino R., Ruggeri M., Zoccante L. (2020). Medically unexplained symptoms in the times of Covid-19 pandemic: A case-report. Brain Behav. Immun. Health.

[23] Greenberg J., Seltzer M., Hong J., Orsmond G. (2006). Bidirectional effects of expressed emotion and behavior problems and symptoms in adolescents and adults with autism. Am. J. Ment. Retard..

[24] Seltzer M., Krauss M. (2001). Quality of life of adults with mental retardation/developmental disabilities who live with family. Ment. Retard. Dev. Disabil. Res. Rev..

[25] Drogomyretska K., Fox R., Colbert D. (2020). Brief Report: Stress and Perceived Social Support in Parents of Children with ASD. J. Autism Dev. Disord..

[26] Colizzi M., Lasalvia A., Ruggeri M. (2020). Prevention and early intervention in youth mental health: Is it time for a multidisciplinary and trans-diagnostic model for care?. Int. J. Ment. Health Syst..

[27] Vivanti G., Kasari C., Green J., Mandell D., Maye M., Hudry K. (2018). Implementing and evaluating early intervention for children with autism: Where are the gaps and what should we do?. Autism Res..

[28] French L., Kennedy E. (2018). Annual Research Review: Early intervention for infants and young children with, or at-risk of, autism spectrum disorder: A systematic review. J. Child Psychol. Psychiatry.

[29] Abir M., Moore M., Chamberlin M., Koenig K., Hirshon J., Singh C., Schneider S., Cantrill S. (2016). Using Timely Survey-Based Information Networks to Collect Data on Best Practices for Public Health Emergency Preparedness and Response: Illustrative Case From the American College of Emergency Physicians’ Ebola Surveys. Disaster Med. Public Health Prep..

[30] Evans J., Mathur A. (2018). The value of online surveys: A look back and a look ahead. Internet Res..

