# Supplementary material for: Psychosocial and Behavioral Impact of COVID-19 in Autism Spectrum Disorder: An Online Parent Survey
Source: Brain Sci. 2020 Jun 3;10(6):341. doi: 10.3390/brainsci10060341 (PMC7349059; doi:10.3390/brainsci10060341)
Supplement: Supplementary file 1 [file brainsci-10-00341-s001.pdf]

Supplementary Table S1. Responses to the open-response question about what medical comorbidity was present in children with ASD.

	**N**	**%**
Neuromotor conditions	55	28.5
Gastrointestinal conditions	48	24.9
Allergies and food sensitivity	45	23.3
Other neuropsychiatric conditions	15	7.8
Rare genetic conditions	9	4.7
Endocrinological and metabolic conditions	8	4.1
Cardiological conditions	6	3.1
Cutaneous conditions	4	2.1
Urinary conditions	3	1.6

Out of 152 respondents, 117 parents reported one medical comorbidity (77%), 31 parents reported two medical comorbidities (20.4%), 2 parents reported three medical comorbidities (1.3%), and 2 parents reported four medical comorbidities (1.3%), for a total of 193 responses.
